# Uc.416 + A promotes epithelial-to-mesenchymal transition through miR-153 in renal cell carcinoma

**DOI:** 10.1186/s12885-018-4863-y

**Published:** 2018-10-04

**Authors:** Yohei Sekino, Naoya Sakamoto, Keisuke Goto, Ririno Honma, Yoshinori Shigematsu, Thang Pham Quoc, Kazuhiro Sentani, Naohide Oue, Jun Teishima, Fumi Kawakami, Jose A Karam, Kanishka Sircar, Akio Matsubara, Wataru Yasui

**Affiliations:** 1 Department of Molecular Pathology, Hiroshima University Institute of Biomedical and Health Sciences, 1-2-3 Kasumi, Minami-ku, Hiroshima, 734-8551 Japan; 2 Department of Urology, Hiroshima University Institute of Biomedical and Health Sciences, Hiroshima, Japan; 3 Cancer Biology Program, University of Hawaii Cancer Center, Honolulu, HI USA; 4 Departments of Translational Molecular Pathology, The University of Texas MD Anderson Cancer Center, Houston, TX USA; 5 Departments of Urology, The University of Texas MD Anderson Cancer Center, Houston, TX USA; 6 Departments of Pathology, The University of Texas MD Anderson Cancer Center, Houston, TX USA

**Keywords:** Uc.416 + A, Renal cell carcinoma, miR-153, Epithelial-to-mesenchymal transition, Sarcomatoid change

## Abstract

**Background:**

The transcribed ultraconserved regions (T-UCRs) are a novel class of non-coding RNAs that are absolutely conserved across species and are involved in carcinogenesis in some cancers. However, the expression and biological role of T-UCRs in renal cell carcinoma (RCC) remain poorly understood. This study aimed to examine the expression and functional role of Uc.416 + A and analyze the association between Uc.416 + A and epithelial-to-mesenchymal transition in RCC.

**Methods:**

Expression of Uc.416 + A in 35 RCC tissues, corresponding normal kidney tissues and 13 types of normal tissue samples was determined by quantitative reverse transcription-polymerase chain reaction (qRT-PCR). We performed a cell growth and migration assay in RCC cell line 786-O transfected with negative control and siRNA for Uc.416 + A. We evaluated the relation between Uc.416 + A and miR-153, which has a complimentary site of Uc.416 + A.

**Results:**

qRT-PCR analysis revealed that the expression of Uc.416 + A was higher in RCC tissues than that in corresponding normal kidney tissues. Inhibition of Uc.416 + A reduced cell growth and cell migration activity. There was an inverse correlation between Uc.416 + A and miR-153. Western blot analysis showed Uc.416 + A modulated E-cadherin, vimentin and snail. The expression of Uc.416 + A was positively associated with the expression of *SNAI1, VIM* and inversely associated with the expression of *CDH1.*

**Conclusions:**

The expression of Uc.416 + A was upregulated in RCC and especially in RCC tissues with sarcomatoid change. Uc.416 + A promoted epithelial-to-mesenchymal transition through miR-153. These results suggest that Uc.416 + A may be a promising therapeutic target.

**Electronic supplementary material:**

The online version of this article (10.1186/s12885-018-4863-y) contains supplementary material, which is available to authorized users.

## Background

Renal cell carcinoma (RCC) accounts for 3% of adult malignancies worldwide [1]. Although there have been improvements in the early diagnosis and surgical treatment of RCC, approximately 30% of RCC tumors are already metastatic at initial diagnosis, and 20–30% of patients who have undergone surgical extirpation will develop distant metastasis [2]. The prognosis of metastatic RCC is poor in part because RCC is often resistant to traditional therapies, such as radiation therapy and chemotherapy. Metastatic RCC may result from epithelial-to-mesenchymal transition (EMT) [3]. Therefore, identifying new molecular mechanisms underlying EMT represents an area of great clinical significance for metastatic RCC patients.

Recently, reports have shown that noncoding RNAs (ncRNAs) are most likely to be essential regulators of the development and progression of RCC [4–6]. Transcribed ultraconserved regions (T-UCRs) are novel class of ncRNAs which are highly conserved among most of the vertebrate genomes [7]. Additionally, T-UCRs are frequently located at both fragile sites and cancer-associated genomic regions [8], indicating that T-UCRs are believed to play critical roles in human cancer. Moreover, it appears that some T-UCRs serve as oncogenes or tumor suppressor genes in some cancers [9, 10]. To date, the interaction between T-UCRs and microRNAs is well-studied with evidence linking T-UCRs with cancer progression. We previously demonstrated that Uc.416 + A was upregulated in gastric cancer and was downregulated in prostate cancer. Uc.416 + A was directly regulated by miR-153 and promoted cancer progression in gastric cancer [11], indicating that Uc.416 + A plays an essential role in cancer progression in some cancer. However, little is known about the expression and biological role of T-UCRs including Uc.416 + A in RCC.

In the present study, we examined the expression of Uc.416 + A in RCC tissues, investigated its functional role of Uc.416 + A in RCC progression and analyzed its involvement in EMT. The current study is the first to investigate the expression and functional role of Uc.416 + A in RCC.

## Methods

### Tissue samples

We used 35 RCC tissue samples (Table 1) for quantitative reverse transcription-polymerase chain reaction (qRT-PCR). The samples were collected from patients at Hiroshima University Hospital or an affiliated hospital. We obtained 15 frozen sarcomatoid RCC tissue samples and adjacent normal kidney samples from the tissue bank of The University of Texas MD Anderson Cancer Center (Houston, TX) after informed consent and using an institutional review board-approved protocol (IRB# LAB 08–670). The clinicopathologic features of this cohort are summarized in Table 2.

**Table 1:** Clinicopathologic characteristics of 35 RCC tissue samples

Number of cases	35
Gender	
M	28
F	7
Median age (years)	64 (55–86)
Race	
Asian	35
Histology	
Clear cell RCC	32
Papillary RCC	3
Pathological T stage	
pT1	5 (14%)
pT2	12 (34%)
pT3	12 (34%)
pT4	1 (3%)
NA	5 (14%)
Pathological N stage	
0	19 (54%)
1	11 (31%)
NA	5 (14%)
Metastasis at time of diagnosis	
0	17 (49%)
1	13 (37%)
NA	5 (14%)

*RCC*: renal cell carcinoma, *NA*: not available

**Table 2:** Clinicopathologic characteristics of 15 RCC tissue samples with sarcomatoid change

Number of cases	15
Gender	
M	7
F	8
Median age (years)	62 (38–76)
Race	
White	11
Hispanic	3
Asian	1
Histology	
Clear cell RCC	11
Papillary RCC	3
Clear cell papillary RCC	1
Mean sarcomatoid component (%)	38 (5–90%)
Pathological T stage	
pT1	1 (6.6%)
pT3	13 (86.6%)
pT4	1 (6.6%)
Pathological N stage	
0	5 (33.3%)
1	5 (33.3%)
Not available	5 (33.3%)
Metastasis at time of diagnosis	
0	5 (33.3%)
1	10 (66.6%)

*RCC*: renal cell carcinoma

### Cell lines

Caki1 (ATCC® CRL-1611) and 786-O (ATCC® CRL-1932) were derived from human kidney cancer (Sumitomo Pharmaceuticals Company, Tokyo, Japan). Both cell lines were maintained as described previously [12]. Both cell lines were tested for mycoplasma contamination by PCR.

### qRT-PCR analysis

Extraction of total RNA, synthesis of cDNA, and qRT-PCR was performed as described previously [13]. Gene-specific amplification of all primers we used in this study was confirmed by a single peak in melting curve analysis (date not shown).

To quantify the level of microRNAs, TaqMan assays were performed as described previously [14]. The expression values were normalized to the expression of the small RNA gene RNU6. miR-153 and RNU6 (Thermo Fisher Scientific, Waltham, MA) were used. The primer sequences and IDs were summarized in Additional file 1**:** Table S1.

### RNA interference

Silencer® Select (Ambion, Austin, TX) against Uc.416 + A was used for RNA interference as described previously [11]. The sequence of siRNA#1 was 5’-GCAUCGCUAUAAUUCAUUAga-3′, and that of siRNA#2 was 5’-GCAUACAUAGCAAAACGAAac-3′. Transfection of cells was carried out with Lipofectamine RNAiMAX (Invitrogen) according to the manufacturer’s instructions.

### Cell growth assay and wound healing assay

To examine cell growth, an MTT assay was performed as described previously [15]. Cell growth was monitored after 1, 2, and 4 days. To examine cell migration, a wound healing assay was performed as described previously [16].

### Western blot analysis

For Western blot analysis, cells were lysed as described previously [17]. Primary antibody. E-cadherin, snail, and vimentin (Cell Signaling Technology, Inc., Danvers, MA) were used. β-Actin (Sigma-Aldrich, St. Louis, MO) was used as a loading control. The IDs and dilution of primary and secondary antibody were summarized in Additional file 2**:** Table S2.

### Transfection of miR-153 mimics

miR-153 mimics (Thermo Fisher Scientific, Waltham, MA) were used for the transfection of miR-153 as described previously [11]. Transfection was performed with Lipofectamine RNAiMAX (Invitrogen) according to the manufacturer’s protocol.

### Luciferase reporter analysis

The sequence of Uc.416 + A which was complementary sequence of miR-153 was subcoloned into the downstream of the luciferase reporter gene in the pGL3-Promoter vector as previously described [11]. The mutant sequence was generated using primer STAR Mutagenesis Basal Kit (TAKARA BIO, Shiga, Japan). For the reporter assay, 786-O cells were plated onto 24-well plates and transfected with pGL3, pGL3-wild Uc.416 + A, or pGL3-Uc.416 + A -mut vector and miR-153 mimics or negative control using Lipofectamine RNAiMAX (Invitrogen). A Renilla luciferase vector pGL 4.75 (Promega Corporation) was co-transfected in order to normalize the differences in transfection efficiency. Luciferase activity was evaluated consequently via Dual-GLO Luciferase Assay System (Promega) following producer’s manual.

### Statistical analysis

Paired T test was used to compare the statistical differences between RCC tissues and their corresponding normal kidney tissues. Statistical differences were evaluated using the two-tailed Student *t*-test or Mann-Whitney *U*-test. *P*-value of < 0.05 was considered statistically significant. Statistical analyses were conducted primarily using GraphPad Prism software (GraphPad Software Inc., La Jolla, CA).

## Results

### Expression of Uc.416 + A is upregulated in RCC tissues

**Fig. 1:**
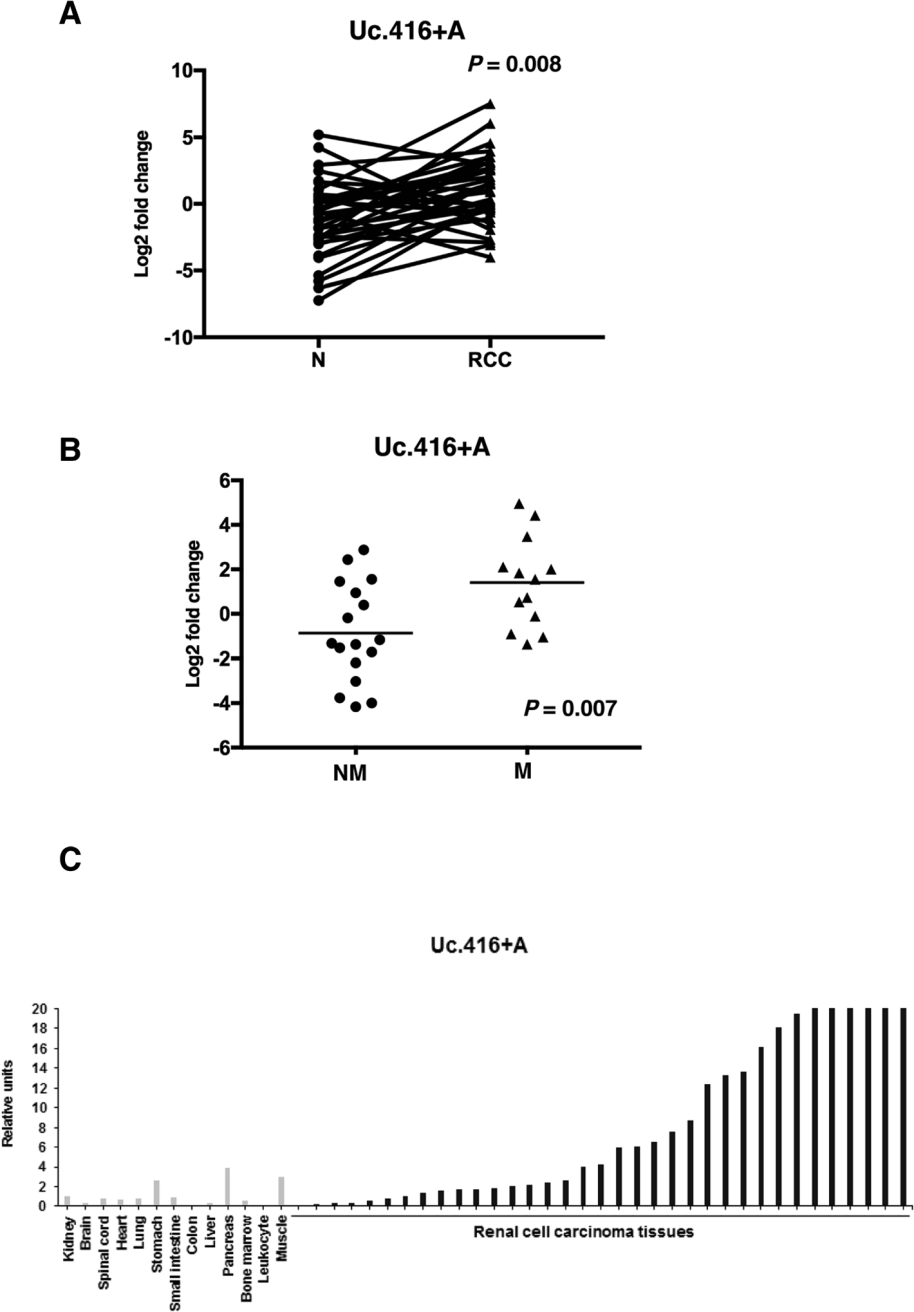
Overexpression of Uc.416 + A in renal cell carcinoma tissues (RCC). **a** The results of qRT-PCR analysis for the expression of Uc.416 + A in 35 RCC tissues and corresponding normal kidney tissues (N). Statistical differences were evaluated with the paired T test. **b** Scatter plot diagrams showing the association between the expression of Uc.416 + A and metastatic status. Statistical differences were evaluated with the Mann-Whitney U-test. NM: non-metastatic RCC tissues. M: metastatic RCC tissues. **c** Results of qRT-PCR analysis for the expression of Uc.416 + A in 35 RCC tissues and 13 types of normal samples

We compared the expression of Uc.416 + A in 35 RCC tissues and corresponding normal kidney tissues by qRT-PCR and considered a RCC tissue/normal kidney tissue ratio > 2.0 as upregulation. As shown in Fig. 1a, expression of Uc.416 + A was upregulated in 71% (25/35) of RCC tissues compared with the corresponding normal kidney tissues (*P* = 0.008). Notably, the expression of Uc.416 + A was higher in metastatic RCC tissues than that in non-metastatic RCC tissues (*P* = 0.007) (Fig. 1b). Then, we examined the expression of Uc.416 + A in 13 types of normal tissue samples and 35 RCC tissues by qRT-PCR. Among these normal tissue samples, the highest expression of Uc.416 + A was found in the pancreas. As expected, 54% (19/35) of the RCC tissues showed higher expression of Uc.416 + A than that in the pancreas (Fig. 1c).

### Knockdown of Uc.416 + A inhibits RCC cell growth and cell migration

**Fig. 2:**
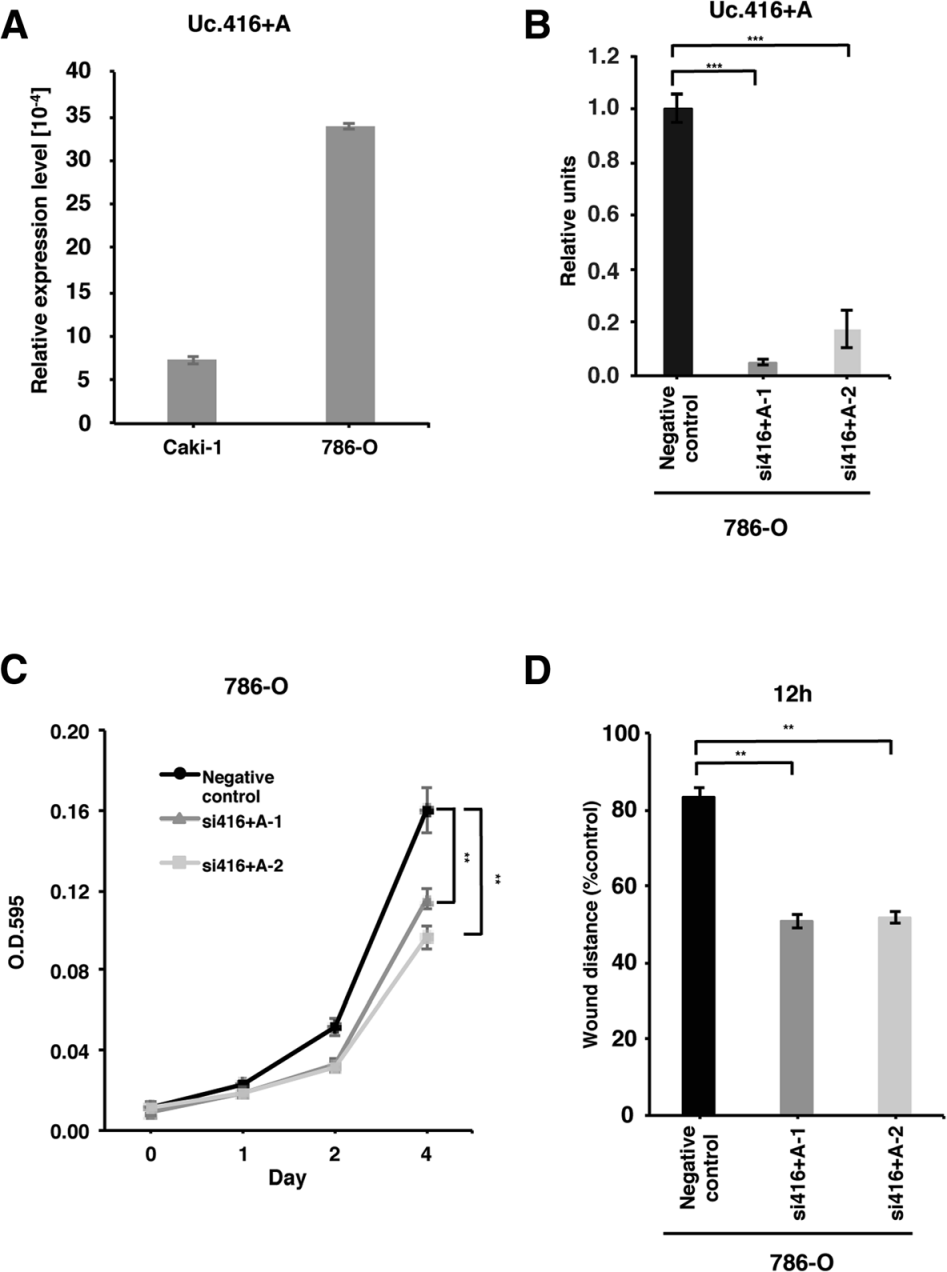
Uc.416 + A is involved in cell proliferation and migration in 786-O cells. **a** The results of qRT-PCR analysis for the expression of Uc.416 + A in 786-O and Caki-1 cells. The results are expressed as the mean ± S.D. of triplicate measurements. **b** The results of qRT-PCR analysis for the expression of Uc.416 + A in 786-O cells transfected with negative control or two different siRNAs. The results are expressed as the mean ± S.D. of triplicate measurements. ***P < 0.001. **c** Cell proliferation assay in 786-O cells transfected with negative control or two different siRNAs. Cell growth was assessed by MTT assays at 1, 2, and 4 days after seeding on 96-well plates. Bars and error bars indicate the mean and S.D., respectively, of 3 independent experiments. **P < 0.01. **d** Wound healing assay in 786-O cells transfected with negative control or two different siRNAs. Wound closures were evaluated by wound contraction percentage and closure time at 0, 12, and 24 h after scratching. The results are expressed as the mean and S.D. of triplicate measurements. **P < 0.01

To further validate the aforementioned findings, we examined the functional role of Uc.416 + A in RCC. We investigated the effects of the downregulation of Uc.416 + A on cell growth and cell migration in 786-O cells showing high Uc.416 + A expression (Fig. 2a). We used siRNA that was specifically designed to target Uc.416 + A and confirmed that the expression of Uc.416 + A was substantially suppressed by treatment with the siRNAs (Fig. 2b). Next, we performed a 4,5-dimethylthiazol-2-yl-2,5-diphenyltetrazolium bromide (MTT) assay and wound healing assay. Knockdown of Uc.416 + A reduced cell growth and cell migration activity (Fig. 2c, d**,** Additional file 3**:** Figure S1).

### Interaction between Uc.416 + A and miR-153

**Fig. 3:**
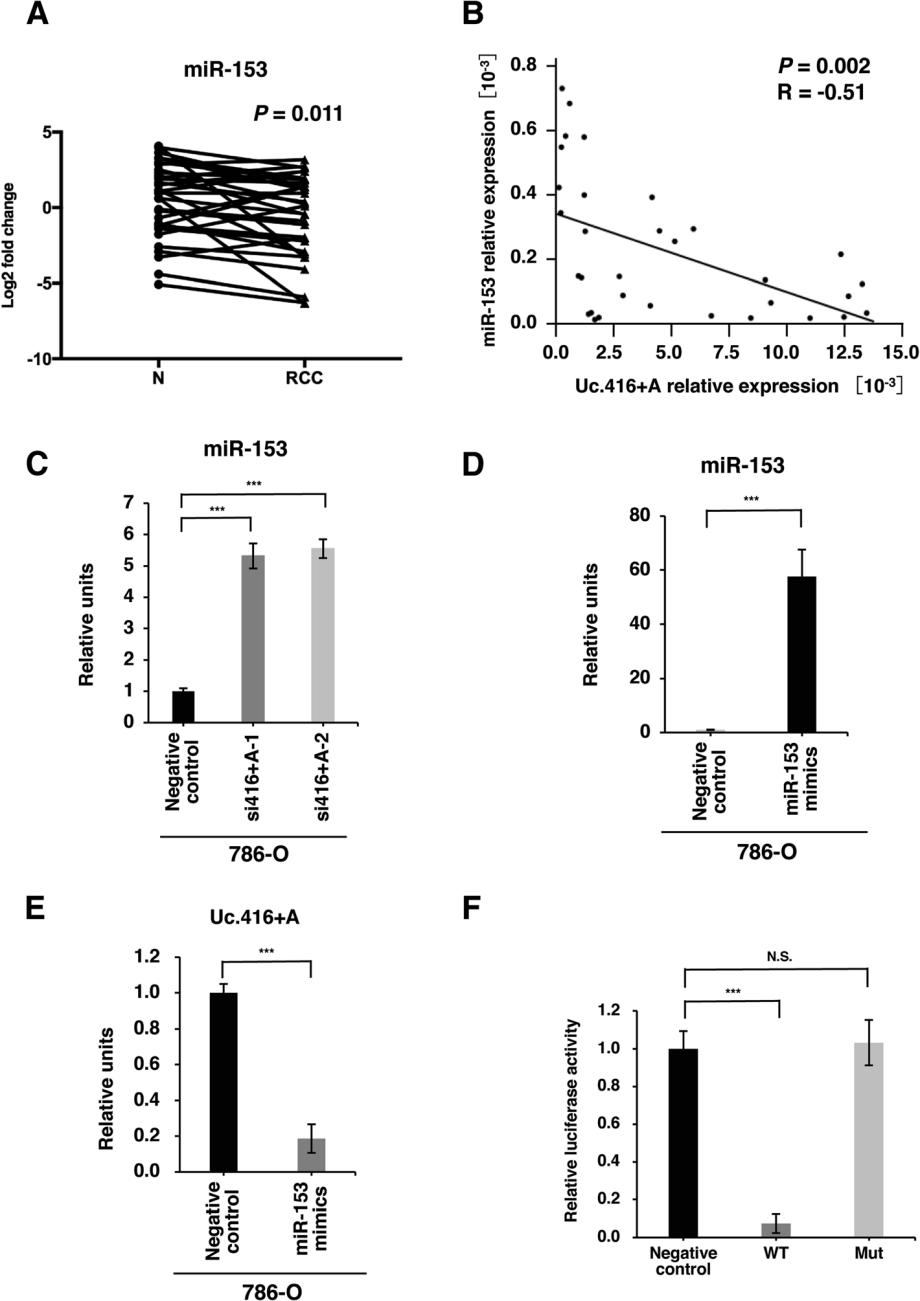
Interaction between Uc.416 + A and miR-153. **a** The results of qRT-PCR analysis for the expression of miR-153 in 32 renal cell carcinoma (RCC) tissues and corresponding normal kidney tissues (N). Statistical differences were evaluated with the paired T test. **b** The correlation between Uc.416 + A and miR-153 in RCC tissues. Spearman correlation coefficient and *P*-values are indicated. **c** qRT-PCR analysis for the expression of miR-153 in 786-O cells transfected with negative control or two different siRNAs. **d** qRT-PCR analysis for the expression of miR-153 in 786-O cells transfected with negative control or miR-153 mimics. The results are expressed as the mean and S.D. of triplicate measurements. ***P < 0.001. **e** qRT-PCR analysis for the expression of Uc.416 + A in 786-O cells transfected with negative control or miR-153 mimics. The results are expressed as the mean and S.D. of triplicate measurements. ***P < 0.001. **f** The luciferase activity of 786-O cells co-transfected with a control pGL3-Promoter vecor containing a wild type Uc.416 + A sequence or a pGL3-Promoter vector containing a mutated Uc.416 + A sequence. The results are expressed as the mean and S.D. of triplicate measurements. ***P < 0.001. N.S., not significant; WT: wild type; Mut: sequence with a mutated miR-153 binding site

Several recent evidence has shown that the interaction between T-UCRs and microRNAs plays essential roles in cancer biology [18, 13]. Previously, we have reported that miR-153 directly regulates Uc.416 + A, which contribute to gastric cancer progression via the regulation of tumor cell growth [11]. We therefore investigated the expression of miR-153 in 32 RCC tissues and corresponding normal kidney tissues by qRT-PCR and considered a RCC tissue/normal kidney tissue ratio < 1.0 as downregulation. The expression of miR-153 was downregulated in 75% (24/32) of RCC tissues compared with the corresponding normal kidney tissues (*P* = 0.011) (Fig. 3a). Although correlation coefficient did not reach 0.7, the expression of miR-153 inversely correlated with the expression of Uc.416 + A (*P* = 0.002, *R* = − 0.51) (Fig. 3b). To further investigate the interaction between Uc.416 + A and miR-153, we examined the effect of Uc.416 + A deregulation on the expression of miR-153. Knockdown of Uc.416 + A upregulated the expression of miR-153 in 786-O cells (Fig. 3c). In contrast, overexpression of miR-153 reduced the expression of Uc.416 + A (Fig. 3d, e). In order to confirm whether similar direct interaction between miR-153 and Uc.416 + A was also seen in RCC, we performed a dual-luciferase reporter assay. The luciferase reporter assay revealed that co-transfection with pGL3-promoter/wild type Uc.416 + A sequence and miR-153 mimics caused a significant decrease in the luciferase activity compared with the negative control. By contrast, the luciferase activity of the mutant Uc.416 + A showed no significant change compared with that of the negative control (Fig. 3f). Collectively, these results indicate that the expression of Uc.416 + A is directly regulated by miR-153 in RCC, which was consistent with a previous evidence found in gastric cancer [11].

### Uc.416 + A stimulates epithelial-to-mesenchymal transition

**Fig. 4:**
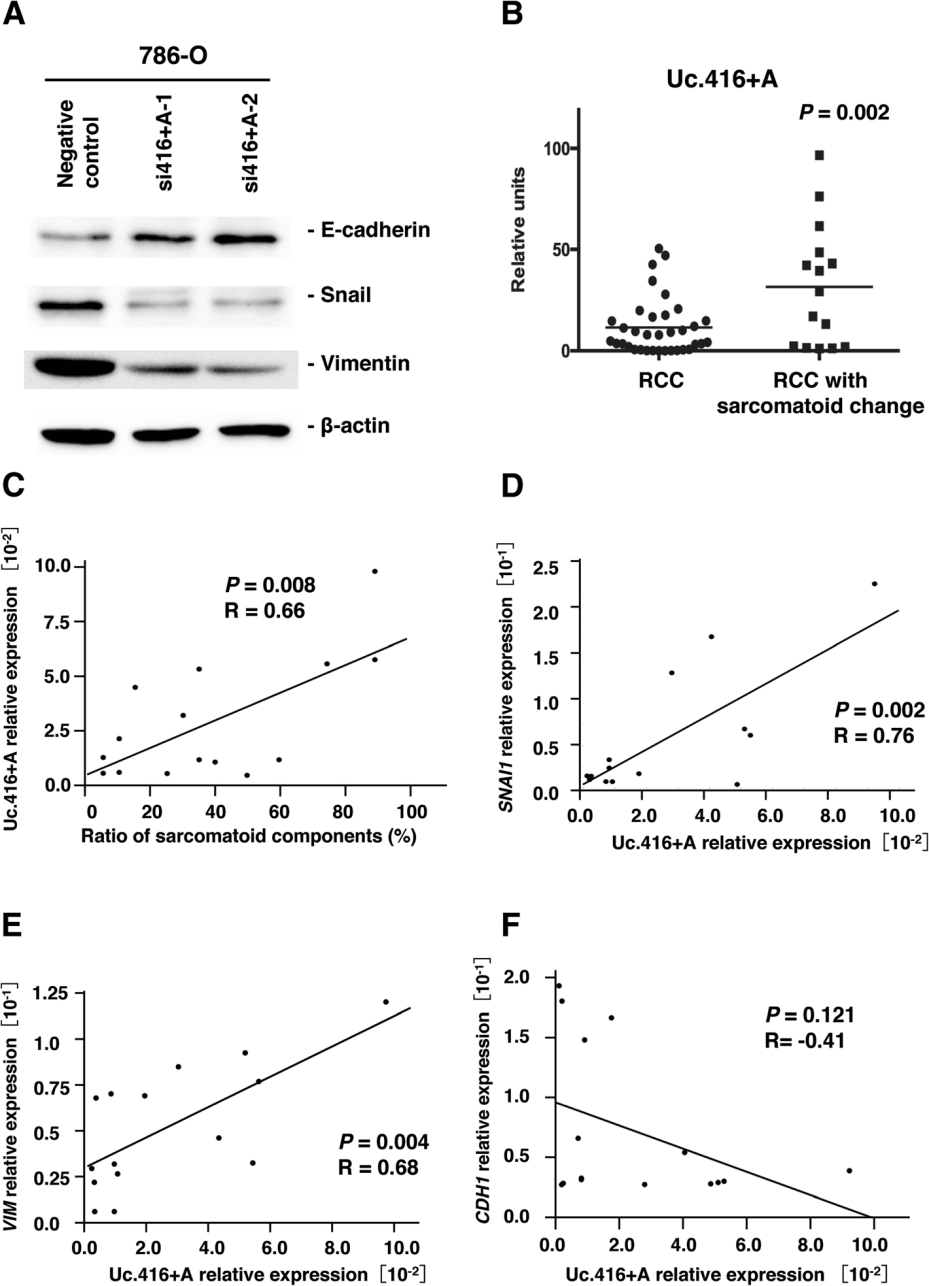
Uc.416 + A modulates epithelial-to-mesenchymal transition. **a** Western blot analysis of E-cadherin, snail and vimentin in 786-O cells transfected with negative control or two different siRNAs for Uc.416 + A. β-actin was used as a loading control. **b** The relative expression level of Uc.416 + A in 35 RCC samples lacking sarcomatoid change and in 15 RCC samples with sarcomatoid change. **c** The correlation between the expression of Uc.416 + A and the ratio of sarcomatoid components in RCC samples with sarcomatoid change. Spearman correlation coefficient and *P*-values are indicated. **d** The correlation between the expression of Uc.416 + A and the expression of *SNAI1* in RCC samples with sarcomatoid change. Spearman correlation coefficient and *P*-values are indicated. **e** The correlation between the expression of Uc.416 + A and the expression of *VIM* in RCC samples with sarcomatoid change. Spearman correlation coefficient and *P*-values are indicated. **f** The correlation between the expression of Uc.416 + A and the expression of *CDH1* in RCC samples with sarcomatoid change. Spearman correlation coefficient and *P*-values are indicated

Several lines of evidence have shown that miR-153 promotes EMT by directly regulating snail [19, 20]. To investigate the effect of Uc.416 + A on EMT, we analyzed the expression of snail, vimentin and E-cadherin in 786-O cells transfected with negative control or siRNA for Uc.416 + A by Western blot analysis. Knockdown of Uc.416 + A reduced the expression of snail and vimentin, and contrarily increased the expression of E-cadherin (Fig. 4a). However, microscopic findings showed that knockdown of Uc.416 + A did not significantly affect cell morphological features (Additional file 4**:** Figure S2). It has been reported that sarcomatoid change often occurs through EMT [21, 22]. We compared the expression of Uc.416 + A in 15 RCC samples with sarcomatoid change and 35 RCC samples without sarcomatoid change by qRT-PCR. The expression of Uc.416 + A was higher in the RCC tissues with sarcomatoid change than that in the RCC tissues lacking sarcomatoid change (*P* = 0.002) (Fig. 4b). There was a positive correlation between the expression of Uc.416 + A and the ratio of sarcomatoid components in 15 RCC samples with sarcomatoid change (*P* = 0.008 *R* = 0.66) (Fig. 4c). To further evaluate the relation between Uc.416 + A and EMT, we examined the expression of SNAI1, VIM and CDH1 in RCC samples with sarcomatoid change and corresponding normal kidney tissues by using qRT-PCR. The expression of Uc.416 + A correlated positively with that of *SNAI1* (*P* = 0.002, *R* = 0.76**,** Fig. 4d) and *VIM* (*P* = 0.004, *R* = 0.68 Fig. 4e). In contrast, although the differences did not reach statistical significance, the expression of Uc.416 + A correlated inversely with that of *CDH1* (*P* = 0.121, *R* = − 0.41, Fig. 4f). Taken together, these findings suggest that Uc.416 + A could potentially effect EMT through the regulation of miR-153.

## Discussion

Several studies have shown that non-coding RNAs exhibit aberrant levels of expression in human cancers, and cancer panels based on their expression profiles have been used to distinguish among distinct cancer types [23, 24]. Although recent studies have clearly linked the expression of T-UCRs to cancers [25, 26], the systematic characterization of T-UCRs in each cancer is not fully understood. In this study, we observed that Uc.416 + A was upregulated in RCC tissues. Our previous study had shown that Uc.416 + A was upregulated in gastric cancer and downregulated in prostate cancer [11]. Therefore, these findings indicate that the expression of Uc.416 + A varies according to the type of cancer. The development of a wide and robust body of experimental evidence on T-UCRs in cancer may lead to more specific and sensitive cancer panels.

Metastatic RCC is often promoted by reactivating EMT in RCC [3]. During the EMT process, epithelial cells lose their cell-cell adhesion and gain a migratory and invasive mesenchymal phenotype [27]. In this study, Uc.416 + A promoted EMT through the regulation of miR-153, which is the first evidence that EMT can be regulated by a specific T-UCR. We also observed that the expression of Uc.416 + A was higher in RCC tissues with sarcomatoid change than that in RCC tissues lacking sarcomatoid change. Given that EMT is though to play an important role in sarcomatoid differentiation [21], our findings suggest that the promotion of EMT through the interaction between Uc.416 + A and miR-153 may be an important mechanism underlying the EMT process and sarcomatoid differentiation in RCC. As sarcomatoid is known to be resistance to standard therapy and associated with poor overall survival in RCC [28–30], patients are encouraged to participate in clinical trials [31]. In this regard, dysregulated elements leading to EMT may offer promising therapeutic targets. In the present study, we showed that the expression of Uc.416 + A was lower in various normal samples than that in RCC tissues, which implies that Uc.416 + A may be a therapeutic target with fewer adverse effects. Accordingly, these novel findings may improve our understanding of sarcomatoid differentiation and aid in the design of a potential treatment strategy in patients with RCC.

In the present study, we observed that Uc.416 + A promoted EMT through miR-153, which may help to explain how Uc.416 + A contributes to cell migration activity in RCC. However, the mechanism whereby Uc.416 + A is involved in cell growth is not fully understood. To date, several microRNA–T-UCR interactions have been identified that lead to tumorigenesis and cancer progression in some cancers [26, 32]. A recent study has reported that Uc.283 + A inhibited pri-miR-195 processing through direct RNA:RNA interaction [18], which is one of the most plausible machineries between miR-153 and Uc.416 + A. Moreover, a recent study has reported that Uc.338 directly regulated the expression of TIMP-1 and promoted metastasis in colorectal metastasis [33], implying that a messenger RNA (mRNA)–T-UCR interaction played an essential role in the cancer. Based on these findings, there could be unknown interactions among microRNA, mRNA, and T-UCR that are potentially regulated by Uc.416 + A. Because the knowledge on T-UCRs as regulators is still in its infancy, further studies are needed to elucidate the regulatory networks related to T-UCRs in cancer.

## Conclusion

Our results showed that Uc.416 + A was overexpressed in RCC, especially in RCC tissues with sarcomatoid change. We also showed that a siRNA target for Uc.416 + A inhibited cell growth and cell migration activity. Furthermore, Uc.416 + A modulated EMT through the regulation of miR-153. Although further studies will be required to clarify how Uc.416 + A contributes to RCC progression, the data presented here highlight the great potential of Uc.416 + A as a therapeutic target in patients with aggressive RCC.

## Additional files

Additional file 1: **Table S1.** Primers sequence for qRT-PCR. (DOCX 15 kb)

Additional file 2: **Table S2.** ID and dilution of primary and secondary antibody. (DOCX 15 kb)

Additional file 3: **Figure S1.** Knockdown of Uc.416 + A reduced cell migration. Representative images of wound healing assays in 786-O cells transfected with negative control or two different siRNAs. (TIF 3180 kb)

Additional file 4: **Figure S2.** Knockdown of Uc.416 + A did not significantly affect the morphological features. Representative microscopic findings in 786-O cells transfected with negative control or a siRNA for Uc.416 + A. (TIF 4598 kb)

## Abbreviations

EMT: Epithelial-to-mesenchymal
MTT: 4,5-Dimethylthiazol-2-yl)-2,5-diphenyltetrazolium bromide
ncRNAs: Noncoding RNAs
qRT-PCR: Quantitative reverse transcription-polymerase chain reaction
RCC: Renal cell carcinoma
T-UCRs: Transcribed ultraconserved regions
