# Supplementary material for: Uc.416 + A promotes epithelial-to-mesenchymal transition through miR-153 in renal cell carcinoma
Source: BMC Cancer. 2018 Oct 4;18:952. doi: 10.1186/s12885-018-4863-y (PMC6172711; doi:10.1186/s12885-018-4863-y)
Supplement: Supplementary file 1 — Table S1. Primers sequence for qRT-PCR. (DOCX 15 kb) [file 12885_2018_4863_MOESM1_ESM.docx]

**Table S1** Primers sequence for qRT-PCR

|  | Forward primer | Reverse primer |
| --- | --- | --- |
| Uc.416+A | CCGCATACATAGCAAAACGA | AGAGCTGCCACTGCCATAAT |
| ACTB | TCACCGAGCGCGGCT | TAATGTCACGCACGATTTCCC |
| SNAI1 | AATCCAGAGTTTACCTTCCAGCA | TCCCAGATGAGCATTGGCAG |
| Vim | CGGGAGAAATTGCAGGAGGA | AAGGTCAAGACGTGCCAGAG |
| CDH1 | GGCTGGACCGAGAGAGTTTC | AATGTACTGCTGCTTGGCCT |

|  | Assay ID |
| --- | --- |
| miR-153-5p | 466540_mat |
| RNU6 | 001093 |
