# Supplementary material for: Uc.416 + A promotes epithelial-to-mesenchymal transition through miR-153 in renal cell carcinoma
Source: BMC Cancer. 2018 Oct 4;18:952. doi: 10.1186/s12885-018-4863-y (PMC6172711; doi:10.1186/s12885-018-4863-y)
Supplement: Supplementary file 2 — Table S2. ID and dilution of primary and secondary antibody. (DOCX 15 kb) [file 12885_2018_4863_MOESM2_ESM.docx]

**Table S2** ID and dilution of primary and secondary antibody

| Primary antibody | ID | Dilution |
| --- | --- | --- |
| E-cadherin | 24E10 | 1:500 |
| Snail | C1503 | 1:500 |
| Vimentin | D21H3 | 1:500 |
| β-actin | A5441 | 1:10000 |

| Secondary antibody | ID | Dilution |
| --- | --- | --- |
| Anti-IgG (H+L chain) (Mouse) pAb-HRP | 330 | 1:500 |
| Anti-IgG (H+L chain) (Rabbit) pAb-HRP | 458 | 1:500 |
