# Supplementary figures and images for: Uc.416 + A promotes epithelial-to-mesenchymal transition through miR-153 in renal cell carcinoma
Source: BMC Cancer. 2018 Oct 4;18:952. doi: 10.1186/s12885-018-4863-y (PMC6172711; doi:10.1186/s12885-018-4863-y)

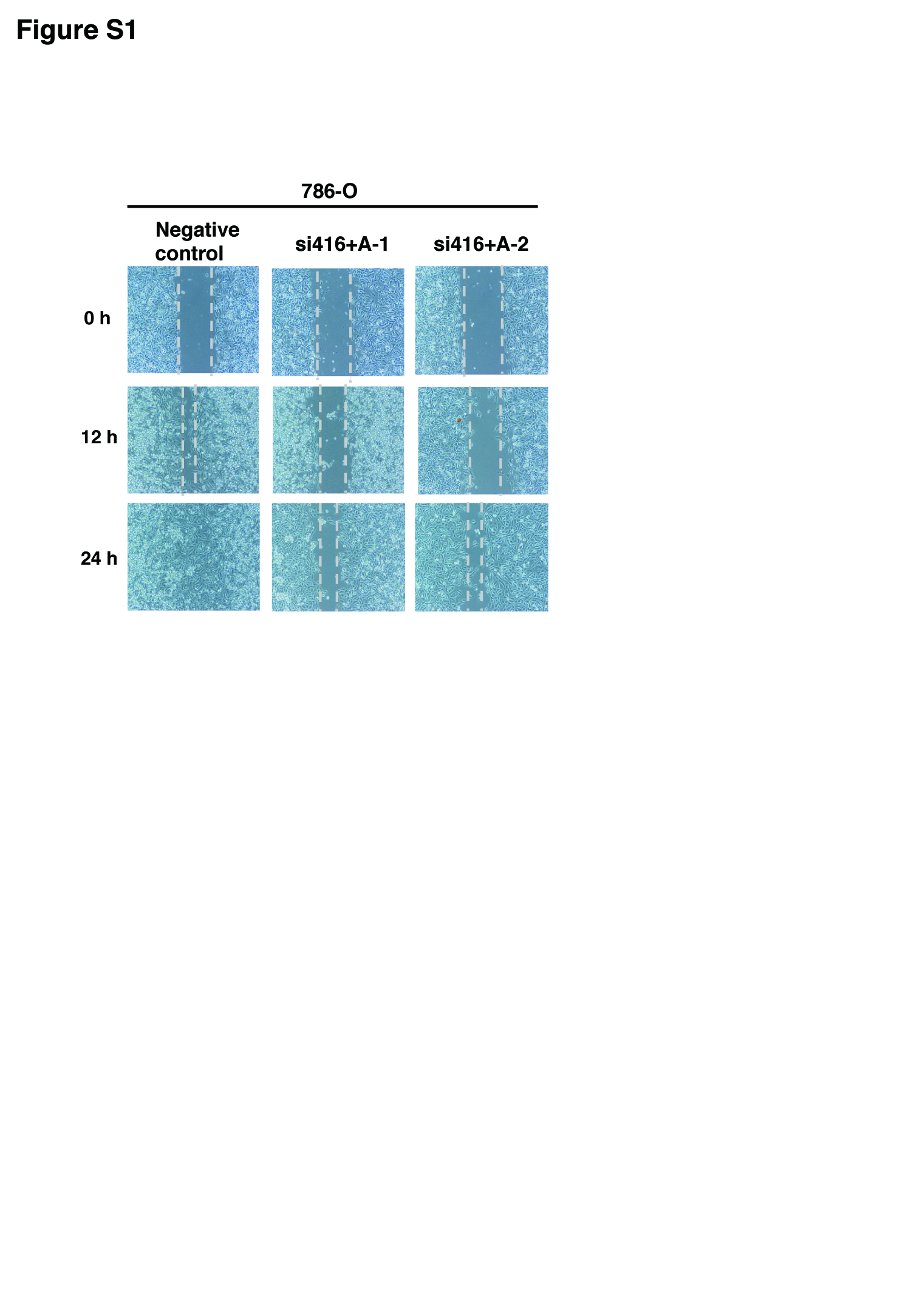

Supplement: Supplementary file 3 — Figure S1. Knockdown of Uc.416 + A reduced cell migration. Representative images of wound healing assays in 786-O cells transfected with negative control or two different siRNAs. (TIF 3180 kb) [file 12885_2018_4863_MOESM3_ESM.tif]

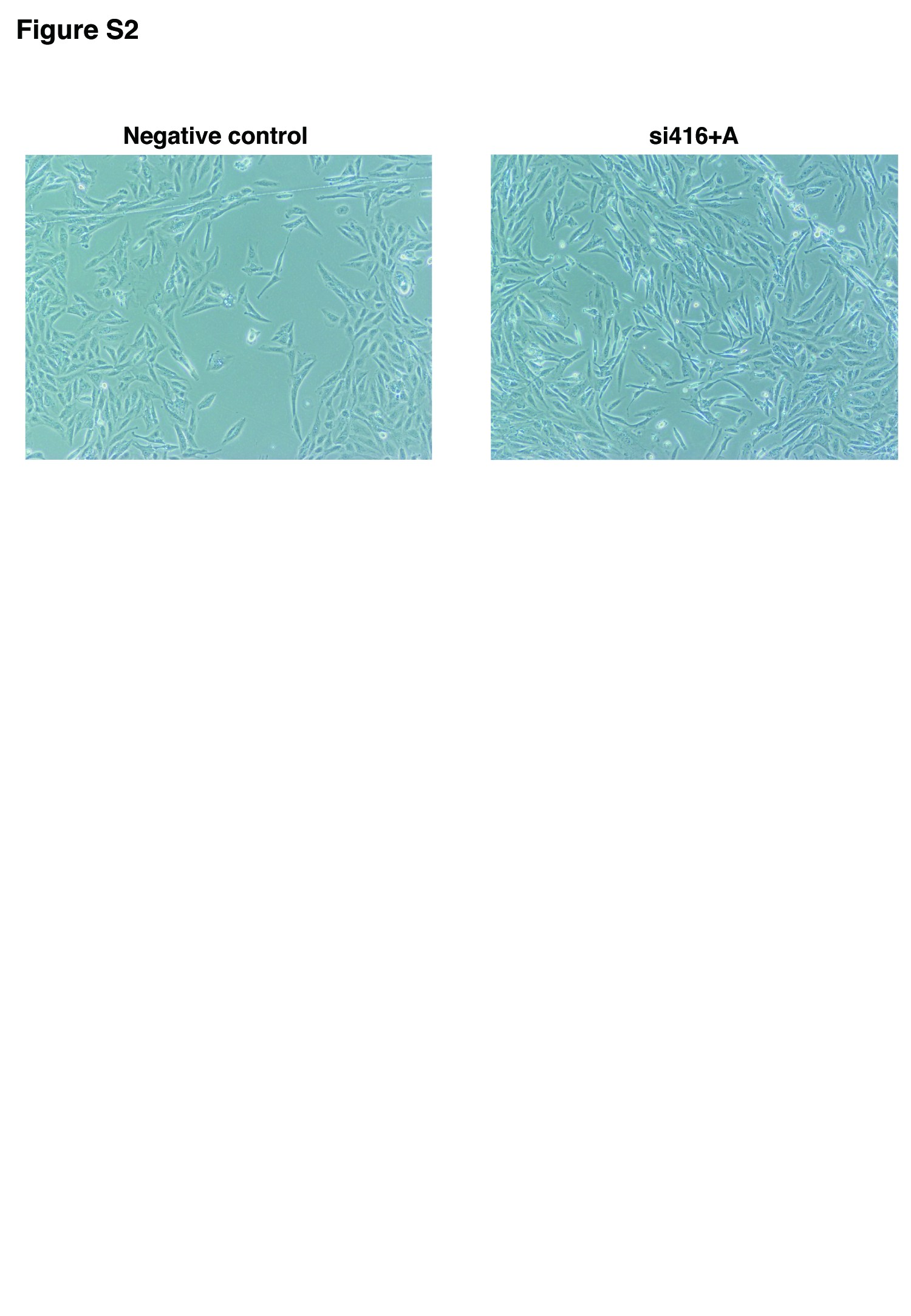

Supplement: Supplementary file 4 — Figure S2. Knockdown of Uc.416 + A did not significantly affect the morphological features. Representative microscopic findings in 786-O cells transfected with negative control or a siRNA for Uc.416 + A. (TIF 4598 kb) [file 12885_2018_4863_MOESM4_ESM.tif]
